# An Account of a Case of Wounded Brain

**Published:** 1807-10

**Authors:** John Syng Dorsey


### *An Account of a Case of Wounded Brain. By* JOHN SYNG DORSEY, M. D.

[ Extracted from the Philadelphia Medical Museum. ]

ON the 19th October, 1805, I was requested to visit. in consultation with Dr. Gardner of Derby, a boy aged about twelve years, who in the morning had received a severe blow from the hoof of horse. As he was seven miles from the city, I did not see him until six hours after the accident had happened. I was informed that he was stunned by the blow, but soon recovering, rose upon his feet and walked; he had not however proceeded far, before he became sick, and fell. He was taken up and carried into the house, which was but a short distance from the place where the accident occurred. A wound was observed upon the back part of the head, just above the left ear, from which some blood flowed, but the quantity lost was inconsiderable. When he was put into bed he became sick, and vomited twice.

At six in the evening I saw him, and found him free from pain, nausea, and coma: he was lively, and did not appear to have suffered much. His pulse was somewhat depressed. Upon examining the head, a wound, an inch and a half long, situated about an inch from the lambdoidal suture anteriorly, exposed to view a large and very deep indentation of the skull; the bone was not thicker than the pasteboard used to cover books, and a consider able portion of it was beat in half an inch below the remaining unbroken portion, so that it was easy to introduce the finger between the skull and dura mater, at this part. Notwithstanding all this injury, there was no nausea, no affection of mind, and very little apparent disturbance to the functions of the patient: there seemed, however, no propriety in suffering the bone to remain depressed, as in all probability, it would have occasioned dreadful consequences.

A free incision was made through the scalp, enlarging very considerably the original wound. The posterior and inferior part of the parietal bone was found broken in pieces, and pressed upon the brain. As there was no opening large enough to admit an instrument to remove the depressed pieces, it became necessary to apply the crown of a trephine to the solid part of the skull. It was applied as near as possible to the fracture, and a segment of bone was removed, forming rather more than a semicircle. This opening enabled me to introduce the levator and to raise the depressed pieces to their proper level. A fragment of bone, very sharp, two-thirds of an inch long, had been forced through the dura mater, and was found imbedded in the brain; a small portion of it projecting upwards, was seized with a pair of forceps; and when it was removed, a considerable discharge of blood followed, but a dossil of lint put an end to the hemorrhage: a soft, light poultice was applied, and an abstinence enjoined from all food, excepting toast and water. Dr. Gardner concurred fully in the propriety of copious depletion, and conducted the patient to a complete and speedy cure. The wound healed without difficulty, and the patient now enjoys good health.

The instances of recovery from wounds of the dura mater are sufficiently rare, to warrant us in recording every encouraging case.

